# Unprecedented Water Effect as a Key Element in Salicyl-Glycine Schiff Base Synthesis

**DOI:** 10.3390/molecules25051257

**Published:** 2020-03-10

**Authors:** Karolina Bakalorz, Łukasz Przypis, Mateusz Michał Tomczyk, Maria Książek, Ryszard Grzesik, Nikodem Kuźnik

**Affiliations:** 1 Department of Organic Chemistry, Bioorganic Chemistry and Biotechnology, Faculty of Chemistry, Silesian University of Technology, B. Krzywoustego 4, 44-100 Gliwice, Poland; Karolina.Bakalorz@polsl.pl (K.B.); Lukasz.Przypis@polsl.pl (Ł.P.); mateusz.tomczyk@polsl.pl (M.M.T.); 2 Department of Physics of Crystals, Institute of Physics, University of Silesia, 75 Pułku Piechoty 1, 41-500 Chorzów, Poland; maria.ksiazek@us.edu.pl; 3 Department of Research and Innovations, Grupa Azoty ZAK S.A. Mostowa 30 A, 47-220 Kędzierzyn-Koźle, Poland; Ryszard.Grzesik@grupaazoty.com

**Keywords:** schiff-base, salen, glycine, hydrolysis, conformation, potassium complexes

## Abstract

Salens, as chelating, double Schiff base ligands, are an important group utilized in transition metal catalysis. They have been used to build interesting functional metal-organic frameworks (MOFs). However, salens interacting with amino acids have also found applications in receptors. Here, we intended to form a “green” glycine-derived salen fragment, but the available literature data were contradictory. Therefore, we optimized the synthetic conditions and obtained the desired product as two different crystallographic polymorphs (orthorhombic Pcca and monoclinic P_21_/c space groups). Their structures differ in conformation at the glycine moiety, and the monoclinic form contains additional, disordered water molecules. Despite the high stability of Schiff bases, these newly obtained compounds hydrolyze in aqueous media, the process being accelerated by metal cations. These studies, accompanied by mechanistic considerations and solid-state moisture and thermal analysis, clarify the structure and behavior of this amino acid Schiff base and shed new light on the role of water in its stability.

## 1. Introduction

**Scheme 1:**
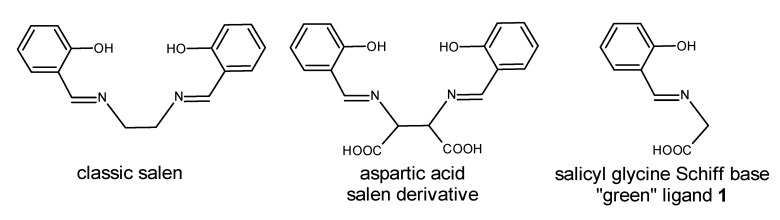
Salen, aspartic acid derived salen, and salicyl-glycine Schiff base **1**.

Schiff bases, due to their conjugation with the aromatic system, constitute an important group of stabilized imines. Salens, which consist of a disalicylic moiety and an aliphatic diamine, have been extensively studied over the past 80 years, resulting in myriad publications [1]. Catalysis is by far the largest application of these compounds [2,3]; more recently, the focus has also been on other features of these systems, including functional metal-organic frameworks (MOFs) [4,5] and anti-cancer agents [6,7]. Salen systems benefit from Schiff base stability, synthetic flexibility, and versatile coordination potential. Although the field of salen synthesis and its resulting properties would seem well-covered, there are still some surprising gaps, which may serve as cornerstones for the next generation of future applications. The relation of salen complexes with amino acids was our particular interest. Salen catalysts have been applied in the synthesis of chiral amino acids [8,9,10]. Another interesting topic is the recognition of amino acids by salen-based receptors [11,12,13,14]. However, the incorporation of amino acids into the salen skeleton remains underdeveloped. Encouraged by the synthetic and functional benefits of the salen systems, we sought to obtain a biodegradable ligand for micronutrient d-block metals. Since a combination of glycine and salicylaldehyde seems to be a perfect candidate for a “green” ligand due to their natural origins, it has received considerable research attention (Scheme 1). However, it was quickly discovered that the literature data was curiously inconsistent; moreover, the degradation of the Schiff base was more rapid than expected. On the other hand the impact of water turned out to be even more surprising. Thus, we present a “green” Schiff base obtained from salicylaldehyde and biogenic glycine as a potential ligand with interesting structural features and transformations.

## 2. Results and Discussion

Our quest for *N*,*O*-ligands led us to literature references suggesting a simple condensation of salicylaldehyde with glycine in ethanol. The synthetic procedures for the desired imine described in the literature differed at several points, and a survey of these procedures is presented in Table 1. To put our experimental findings into context, the details of the literature procedures are compared below. Only one reference described a procedure without the addition of a base [15]. In this method, glycine was dissolved in 20 mL of water and salicylaldehyde in 10 mL ethanol. These solutions were mixed and refluxed for 3 h while turning orange. The workup was as follows: the volume was reduced to half, and the desired product crystallized upon cooling; the product was filtered off, washed with ethanol and ether, and finally dried. The crude product was recrystallized with an ethanol-propanol mixture (60:40) to give the desired product with a 66% yield. In the Zhu synthesis [16], similar reactant solutions were used (methanol was used instead of ethanol), but the aqueous solution was alkalized with KOH. In addition, shorter reaction time and room temperature were used. The product remained in solution and was not separated, so the yield was not specified. Gürkan performed the reaction [17] at low temperature (10 °C) under an N_2_ atmosphere. Contrary to Zhu, NaOH was used as the base. After 2 h, the solid product was formed and recrystallized with methanol-ether (1:1) to give, after filtration, the ligand with a 58% yield. Panchal was the first to publish a synthetic method that avoided water [18]. He carried out the reaction in ethanol using a stoichiometric addition of KOH at 50 °C for 1 h. The product was crystallized by diethyl ether diffusion giving a product with 63% yield. In a similar way, Zhao [19] performed his synthesis at room temperature but did not specify a reaction time. The yield and separation methodology were the same as in the Panchal method.

**Scheme 2:**
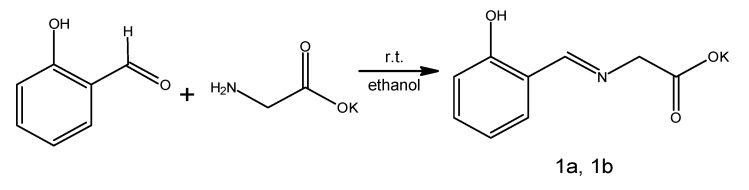
Synthesis of **1**—potassium *N*-[(2-hydroxyphenyl)methylene]glycinate.

However, despite many attempts, we were unable to repeat any of the five procedures listed in Table 1, although these approaches consist of classical methods of Schiff base synthesis with precipitation of the desired product often in a fine crystal form. Finally, after modifying the conditions, we obtained the desired product **1**—potassium *N*-[(2-hydroxyphenyl)methylene]glycinate. It is worth noting that we identified the factor determining success. Initial efforts focused on obtaining the non-salt product. Chohan’s conditions [15] were adopted; however, the results achieved differed from the published report. Separation of an orange, oily, residue on the bottom of the flask was observed. After reducing the volume and cooling, a solid product was not obtained. After 24 h, a few single crystals appeared, which, according to NMR, corresponded to the expected product but with a large excess of glycine. Because the attempt was unsuccessful, we modified the reaction conditions. Changing the solvent to ethanol resulted in the incomplete dissolution of glycine, as expected. During the reaction, the mixture changed to yellow, and glycine gathered at the bottom of the flask. After 3 h of heating, the remaining glycine was filtered, and the filtrate evaporated. A solid product was not obtained, and further analysis did not confirm the expected product. The reaction was carried out using a water:ethanol mixture (1:2), but results similar to those using an excess of water were obtained. Another solvent mixture (5 parts water to 1 part ethanol) was used just to dissolve the aldehyde. The mixture again turned yellow with significant solid formation; though, regrettably, NMR analysis showed that it was only glycine. After performing the above-described approaches no satisfying experimental results were obtained. Our observations led to the conclusion that, on the one hand, water disables separation of the expected product, while, on the other, dissolution of glycine is difficult in alcohol only. Conversion of glycine into a salt allows its dissolution in alcohol. Thus, we turned our efforts to obtaining the product in salt form by adopting the Zhao conditions [19]. Equimolar alkalization of glycine with KOH enabled the formation of a salt that was soluble in ethanol, allowing us to obtain a homogenous mixture with salicylaldehyde. Upon its addition, the mixture changed color immediately to an intense orange with the immediate formation of an orange precipitate removing the need for diethyl ether addition for product separation. The solid was filtered off and air-dried. During this time, we observed in the bulk solid an exceptionally interesting process of color transformation from an intense orange to a bright yellow. The initial yield of the reaction was 84%; however, NMR analysis indicated contamination with the substrates. The reaction was optimized, and after a few trials (using methanol as a solvent), the pure product was produced with a final yield of 74% (Scheme 2).

Spectroscopic data were initially consistent with our expectations and unambiguously confirmed by X-ray crystallography; though, curiously, the spectroscopic data obtained did not agree with the literature references and the overall observations during the synthesis.

However, it was observed that the freshly formed orange crystals (**1a**) rearranged within 1–2 days in the air into a new, yellow form (**1b**). This observation was independent of the scale. Crystals of both forms were collected and analyzed by X-ray diffraction. Freshly crystallized crude product **1a** formed an orthorhombic Pcca lattice. On the other hand, the yellow crystals **1b** crystallized in the monoclinic P2_1_/c space group.

**Figure 1:**
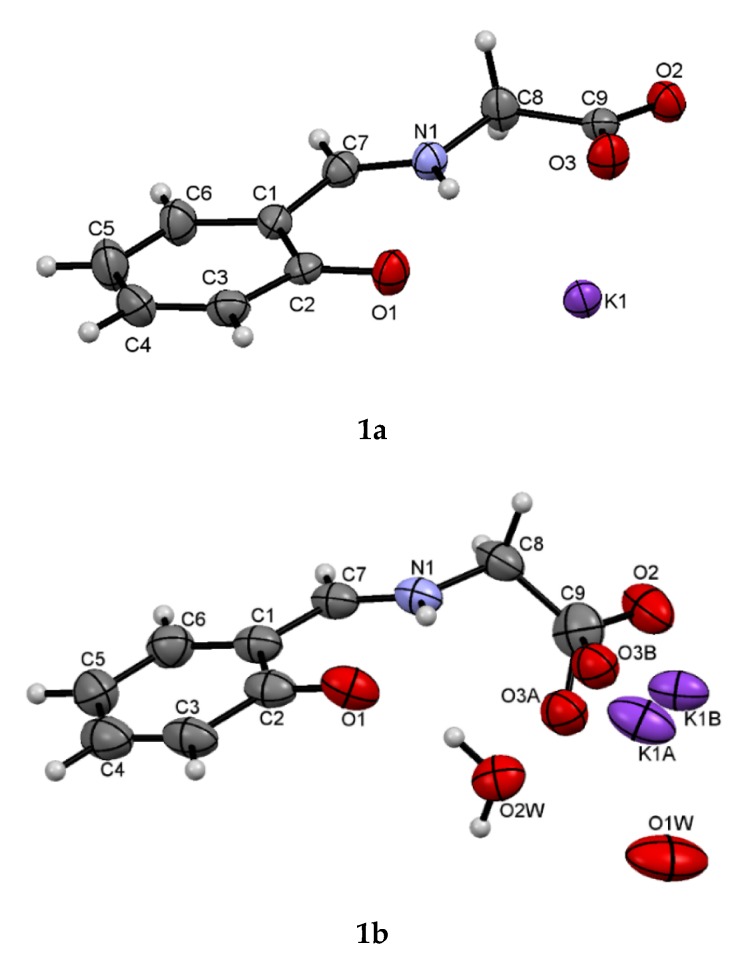
Molecular structures of compounds **1a** and **1b** showing the atom-labeling scheme. Displacement ellipsoids are drawn at the 50% probability level.

The chemical composition of both crystalline forms is similar—**1a** represents pure compound **1** without additional solvent molecules, while **1b** contains additional disordered water molecules. It is worth noting that the oxygen atom O3 from the main residue (carboxyl group) and the potassium ion are also disordered (Figure 1). However, the structural differences between the two crystals are more significant. In order to determine the conformation of the two forms, the torsion angles C7-N1-C8-C9 were analyzed. For form **1a**, its value of 172.39(18)° corresponds to the antiperiplanar conformation along the N1-C8 bond. In the case of form **1b**, the angle mentioned is equal to 124.46(44)°, corresponding to the anticlinal conformation. This conformation is present in nickel complexes with substituted glycine of the same Schiff base skeleton; however, the torsions fall between 106.36–112.09° [20,21]. On the other hand, copper complexes with similar ligand derivatives form synclinal conformations with a range from 58.85–72.27° for their torsion angles [22]. Nevertheless, none of the reported structures of the glycine-salicylaldehyde imine derivatives forms the antiperiplanar conformation as found in the metastable structure **1a**.

Protonation of the nitrogen atom N1 in both structures results in an N1-C7 bond length of 1.287(2) in **1a** and 1.301(5) Å in **1b**, similar to the standard value [23].

**Figure 2:**
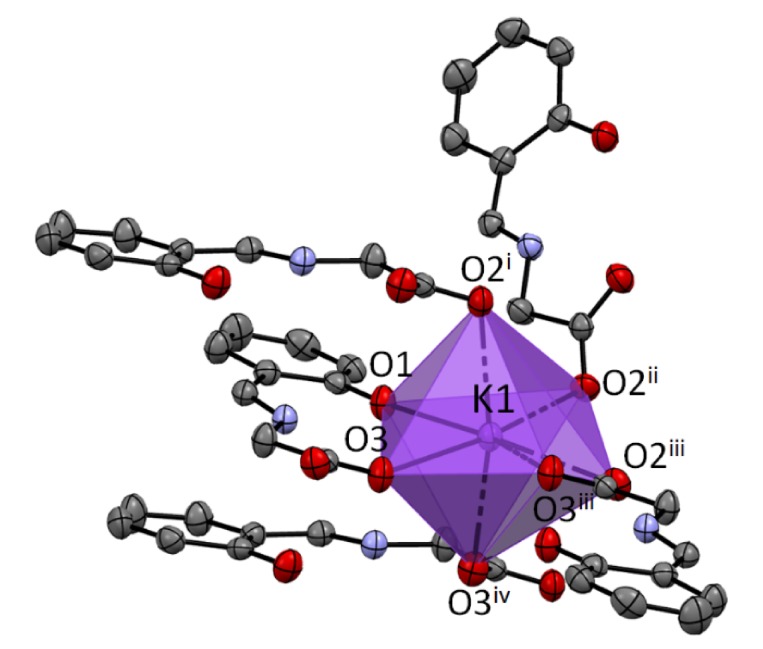
Packing diagram showing the seven-coordinate geometry of the K ion in **1a** (i: 1 − x, y, ^5^/_2_ − z; ii: ½ + x, y, 2 − z; iii: 1 − x, −y, 2 − z; iv: 1 − x, y, ^3^/_2_ − z).

The coordination number of potassium is 7 for the structure **1a**. The K ion is bonded to one O1, three O2, and three O3 atoms. It creates a very regular 1:4:2 7-coordinate geometry with only the O1 atom and one O3 atom in a unique position (Figure 2).

In **1a**, the parallel arrangements of the neighboring aromatic rings with the common potassium cations are most probably a consequence of their π-stacking. Intermolecular interactions in **1b** do not reveal π-stacking of the aromatic systems, but consist of bonding to the carboxyl groups.

In both structures **1a** and **1b,** intramolecular hydrogen bonds are present (Table 2). The structure **1a** is stabilized by two hydrogen bonds between the N1 atom and the oxygen atoms O1 and O3. According to the characteristics of hydrogen bonds proposed by Desiraju and Steiner [24], they can be classified as strong, conventional bonds. Structure **1b** is also stabilized by an intramolecular hydrogen bond with parameters corresponding to strong, conventional bonds. It is worth noting that no hydrogen bond between N1 and the oxygen atoms from the disordered carboxylic group is observed, although a weak intermolecular hydrogen bond between C8 and the O3A atom from the carboxylic group occurs. There are also weak hydrogen bonds involving the water molecule with the O2W atom. Table S1 contains detailed geometric parameters of the hydrogen bonds present in both structures. The characteristics of the fingerprint plots and the Hirshfeld surface are given in the Supplementary Material.

**Scheme 3:**
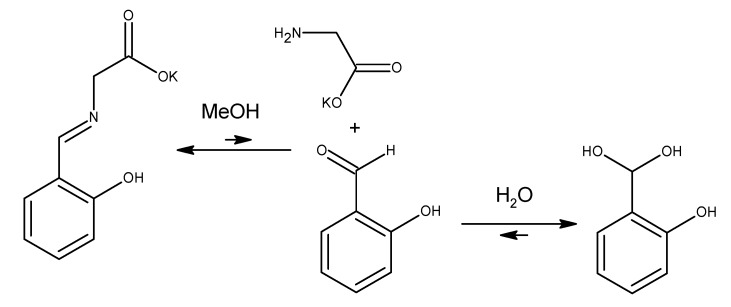
Hydrolysis of 1 with a consecutive equilibrium.

**Figure 3:**
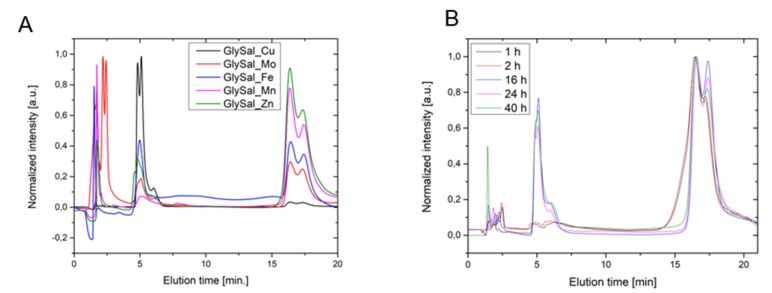
Hydrolysis progress chromatograms of (**A**) reactions of ligand **1** with metal ions, (**B**) ligand **1** water solution.

Synthesis of the Schiff base **1** in the presence of water was not successful; however, encouraged by reports on the formation of metal complexes with ligand **1** using a variety of transition metal ions (Cu, Co, and Mn), we thought that it was still possible to synthesize them, as metal complexes tend to be more stable structures [15]. Thus, complexation of Cu, Zn, Fe, Mn, and Mo ions with ligand **1** in an equimolar ratio (see experimental section for details) in water was performed. During these reactions, the formation of colored mixtures was observed, potentially indicating successful complexation. To confirm the formation of these new products, the reaction mixtures were analyzed by HPLC using MeOH:H_2_O (30:70) as the eluent. All the chromatograms showed evidence of polar products between 0–4 min (metal sulfates, glycine, and salicylic acid) and a peak at 5 min, the area of which was proportionally reversible to a broad peak between 15–20 min corresponding to salicylaldehyde (Figure 3). The peak appearing around 5 min was presumed to be the polar metal complex of **1**, but the presence of salicylaldehyde in different amounts indicated hydrolysis. These observations led us to assess the behavior of **1** in water over time. In this experiment, we observed that ligand **1** in water decomposed mostly to salicylaldehyde, glycine, and the substance, with a retention time of around 5 min. As the concentration of free amino acid increased, the intensity of the peak at 5 min also increased, allowing us to recognize this signal as most probably salicylaldehyde hydrate, which forms under the basic conditions present in the aqueous solution (Scheme 3). The peak position can shift and depends on the amount of the base added.

We performed UV-Vis experiments to confirm our observation on the hydrolysis of compound **1** during HPLC and determined the rate of this phenomenon. We tested the behavior of compound **1** in methanol (HPLC grade) with the addition of water in various ratios. We also added separately Fe^3+^ or Cu^2+^ to assess whether these metals would form complexes or catalyze hydrolysis. Additionally, we took UV-Vis spectra of salicylaldehyde and glycine in methanol with the addition of KOH and the abovementioned salts in order to study the stability of the reactants and the formation of the complexes potentially formed after the eventual hydrolysis. The addition of 0.1 equivalent of Fe^3+^ or Cu^2+^ to the methanolic solution of **1** (Figures S5 and S6) caused the absorbance at 400 nm to decrease immediately (in less than 5 min). This band, which is present in the spectrum of compound **1**, is specific to the C = N imine bond. It did not disappear completely because in this region there is also a band of salicylaldehyde hydrate, which was identified by its spectra with the addition of KOH (Figure S7). For this reason, during the experiment with the addition of one equivalent of water to compound **1**, we did not observe any significant decrease in the absorbance (Figure S12) because of the equilibrium between these compounds. Only 10 times more water made a change to this band (Figure S13). The comparison of the spectra of the Fe-salicylaldehyde and Fe-glycine complexes to the mixture of **1** with Fe^3+^ (Figure S10) may indicate that after hydrolysis we also observed the formation of a complex of the aldehyde, which can confirm the hydrolysis. These results are supported by the HPLC observation that imine **1** hydrolyzes rapidly (within 5 min) in the presence of a metal, and therefore the complex of **1** cannot be obtained in water.

**Scheme 4:**
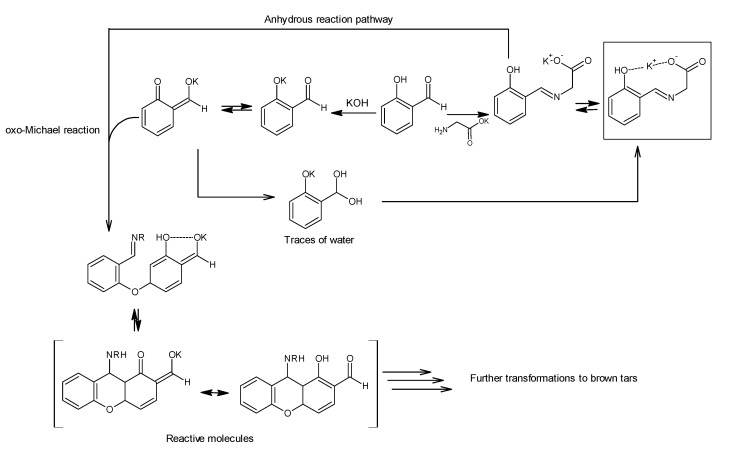
Proposed reaction pathways.

Despite the general high stability of Schiff bases, it was found that this particular base undergoes hydrolysis in aqueous solutions at room temperature. Moreover, this reaction is followed by consecutive condensations, which most likely lead to polymeric, dark products within several days. Additionally, it is important to note that any attempts to synthesize transition metal complexes (such as Fe, Cu, and Mn) of **1** in aqueous media failed due to the accelerated hydrolysis of the imine. However, stable complexes were obtained from a non-aqueous, aprotic environment such as DMF (see Supplementary Material for synthetic details ), but their contact with water still led to hydrolysis products. Based on the above facts, it can be concluded that the presence of water has a destructive effect on the ligand and leads to hydrolysis. There were two crystallographic structures of compound **1**, which differed in the content of water in the crystallographic unit cell. Therefore, to get a better idea of the chemical nature of this compound, additional experiments were performed. Literature reports indicated that metastable Schiff bases were obtained by the solvent-free method, thus limiting the adverse effect of water on the reaction balance [25,26,27]. Similar experiments were undertaken “without water” as follows: glycine and potassium hydroxide were stirred, and freshly distilled salicylaldehyde was added slowly. An emulsion formed which, while stirring, began to intensively heat up and change color. This reaction was carried out at 70 °C. After 30 min, we still observed large amounts of salicylaldehyde (conversion monitored by TLC). After 1 h, we quenched the reaction, and the reaction mixture was worked up to give a metastable oil-solid system. In addition, TLC analysis showed the presence of many new, inseparable products. A ^1^H-NMR of this mixture confirmed our assumptions that we had obtained a very complicated mixture in which many signals were observed in the aromatic range (see Figure S7). To clarify the above observations, we hypothesized that trace amounts of water present in methanol led to the desired product, whereas an excess of water led to hydrolysis and, in its absence, other, different side reactions occurred. It is probable that under these anhydrous conditions, as the next step of condensation, the resulting ligand reacts with salicylaldehyde in an oxo-Michael reaction to form a chromanone derivative which can further react with compounds present in the mixture (Scheme 4) [28,29,30,31,32].

Additionally, we performed experimental studies on the thermal stability of both forms **1a** and **1b** in their solid states and the reversibility of their transformation to each other. Thus, the obtained product from the reaction containing both forms **1a** and **1b** was dried on a moisture analyzer to remove water (Figure S16), leading to an intense orange compound, which was further analyzed by TGA under an argon atmosphere (Figure S15). No weight loss that could be assigned to water was detected up to 200 °C. At this temperature, decomposition of the compound started; thus, we assume that it was the pure **1a** form of the Schiff base. Furthermore, the **1a** form was exposed to moisture in a phytotron chamber (see SI for the details) until the whole volume of the compound turned light yellow, suggesting the exclusive formation of the **1b** form. Measurement of the moisture content for this form of compound **1** with a moisture analyzer showed that 7.69% of the mass was water, which almost corresponds to its stoichiometric content of 7.65%. Additionally, TGA analysis of the **1b** form showed a 7% weight loss in the temperature range from 25 °C to 100 °C, which can be assigned to the removal of water, while above this temperature the course of **1b**’s decomposition was identical with **1a**, confirming that both compounds are chemically identical. The difference in water content between those two analyses may be connected with the transformation of **1b** into the more stable **1a** form, as confirmed by crystallographic structures, during the few hours that it took us to transport the sample for TGA analysis, despite our efforts to preserve the **1b** compound unchanged. Finally, we dried the **1b** form to **1a**, and the course of the TGA decomposition was identical with the original **1a** form, confirming the full reversibility of the hydration process and the lack of any negative influence on both structures.

## 3. Conclusions

Based on the solid-state studies, the behavior of the Schiff base (**1**) in solution, and the investigations on its synthesis, we can summarize our results as follows: Potassium *N*-[(2-hydroxyphenyl)methylene]glycinate (**1**) could be successfully synthesized with high yields in basic conditions in methanol. The spectroscopic data agree with the structure proven by X-ray analysis. The overall analysis reveals the presence of two crystallographic forms differing in color (yellow and orange), crystallographic system, and degree of hydration. Traces of water present in the reactants (KOH and technical grade methanol) are beneficial for obtaining the product. However, in alcoholic solution, one equivalent of water with respect to compound **1** leads to its hydrolysis. This process is additionally accelerated by the presence of Lewis acids such as Fe^3+^ or Cu^2+^. On the other hand, Cu complexes with ligand **1** could be obtained in non-aqueous media.

## 4. Materials and Methods

Glycine (5.0 g, 67 mmol) and one equiv. of KOH (3.7 g, 66 mmol) were dissolved in methanol (50 mL). A solution of salicylaldehyde (8.1 g; 66 mmol) in methanol (50 mL) was added. The color of the solution rapidly turned yellow with the formation of an orange precipitate. The mixture was stirred for 1 h at room temperature. The resulting solid product was filtered and air-dried. During drying, the color of the product changed from orange to yellow. Yield 74%, (10.7 g, 49 mmol) m.p. = 219–221 °C.

NMR spectra were recorded on an Agilent 400 MHz spectrometer at room temperature. Aromatic signals were assigned by simulations using ChemDraw^®^ software (license No. 2013781467484).

^1^H-NMR (400 MHz, CD_3_OD) δ 8.34 (s, 1H, C_Ar_-CH=N), 7.30 (d, *J.* = 7.6 Hz, 1H *o*-C_Ar_-H), 7.27 (dd, *J.* = 8.2, 7.4 Hz, 1H, *p*-C_Ar_-H), 6.79 (dd, *J.* = 8.2, 0.8 Hz, 1H, *m-*C_Ar_-H-C_Ar_-OH), 6.73 (ddd, *J.* = 7.6, 7.4, 0.8 Hz, 1H, *m-*C_Ar_-H-C_Ar_-H), 4.23 (m, 2H, =N-CH_2_). ^13^C-NMR (400 MHz, CD_3_OD) δ 175.80 (COO), 168.00 (CH=N), 167.92 (C_Ar_-OH), 134.85 (*p-*C_Ar_-H), 133.61 (*o*-C_Ar_-H), 119.78 (*m*-C_Ar_-H), 119.17 (C_Ar_-CH=N), 117.88 (C_Ar_-H-C_Ar_-OH), 60.89 (=N-CH_2_). MS for C_9_H_9_NO_3_ [M+H]^+^: calculated = 180.1; found: 180.2.

### 4.1. Attempted Syntheses of Cu, Zn, Fe, Mn, and Mo. Metal. Complexes with Ligand 1

HPLC analyses were carried out using a Dionex UltiMate 3000 HPLC system equipped with an autosampler. Chromatographic separation was achieved with Thermo Scientific Hypersil GOLD HPLC (125 × 3 mm; 5 μm particle size) column, using water (solvent A, 70%) and methanol (solvent B, 30%). Sample injection volumes were 10 μL, the flow rate was 1 mL/min, and the column temperature was maintained using an oven at T = 23 °C.

In a 50 mL round bottom flask, 10 mL of a 1 mM solution of ligand **1** was mixed with 10 mL of a 1 mM solution of metal sulfate and stirred for 1 h. 20 μL was then taken and diluted with distilled water to 2 mL, filtered through a 0.22 μm pore size syringe filter, and analyzed by HPLC.

The synthesis of the Cu complex with **1** is described in the Supplementary Materials.

### 4.2. Hydrolysis Experiment

In a 25 mL round bottom flask, 1 mmol of ligand **1** was dissolved in 10 mL of water and stirred for 40 h. After 1, 2, 16, 24, and 40 h, a 20 μL solution aliquot was diluted with distilled water to 2 mL, filtered through a 0.22 μm pore size syringe filter, and analyzed by HPLC.

### 4.3. UV-Vis. Measurements

UV-Vis analyses were carried out using a Hitachi U-2910 in the wavelength range 190–900 nm. 10 mm quartz cuvettes were used. Sample preparation: 10 mL of a 1 mM solution of ligand **1** in methanol (HPLC grade) was prepared, and then one equivalent or 10 equivalents of water was added, respectively. These solutions were transferred sequentially to the cuvette, capped, and measured for at least 6 h. 10 mL of a 1 mM solution of ligand **1** in methanol (HPLC grade) was then measured as a blank, followed by the addition of 0.1 and 1 equivalent FeCl_3_ or CuCl_2_, respectively, which were measured for at least 3 h. Additionally, the following solutions were prepared and measured: 1 mmol solution of salicylaldehyde + 1 equiv. KOH in methanol followed by the addition of 1 equiv. of FeCl_3_; and 1 mmol solution of glycine + 1 equiv. KOH + 1 equiv. FeCl_3_. All UV-Vis spectra are attached to the supplementary material.

### 4.4. X-ray Diffraction Measurements

X-ray diffraction data were collected using an Xcalibur diffractometer with Mo Kα radiation (λ = 0.71073 Å) at room temperature for crystal **1a** and using a SuperNova diffractometer with Cu Kα radiation (λ = 1.54184 Å) at 120 K for crystal **1b**. Data were integrated with CrysAlis^Pro^ software (version 1.171.39.15e for 1a, 1.171.38.41q for 1b) [33]. The phase problem was solved by direct methods with SHELXS-97 [34]. The model parameters were refined by full-matrix least-squares on F^2^ using SHELXL-2018/3 [34]. All programs were included in the WINGX package (version 2018.2) [35].

**1a** represents a pure compound without any additional solvent molecules. Compound **1b** is a hydrate and is also a pure compound. The presence of the additional atoms results from the disordering of the carboxyl group and the K+ ions. The number of water molecules can be defined based on an analysis of the occupancies of the oxygen atoms in the water molecules. The O1W atom from the first water molecule has an occupancy equal to 0.5. This atom is placed in the special position (the coordination is 0.5, 0.5, 0.5), and thus the real occupancy is 1. The O2W atom from the second water molecule has an occupancy equal to 0.46(1). In the final refinement, it was fixed at 0.5. The hydrogen atoms that are present at the O2W atom were found on the difference Fourier map. In order to preserve the correct geometry of the water molecules, the hydrogen atoms were placed using a suitable HFIX. In the case of the O1W atoms, the hydrogen atoms were not visible on the difference Fourier map.

CCDC 1957836-1957837 contains the supplementary crystallographic data for this paper. These data can be obtained free of charge via http://www.ccdc.cam.ac.uk/conts/retrieving.html (or from the CCDC, 12 Union Road, Cambridge CB2 1EZ, UK; Fax: +44 1223 336033; E-mail: deposit@ccdc.cam.ac.uk).

Crystal Data for **1a** (C_9_H_8_NO_3_K; M = 217.26 g/mol): orthorhombic, space group Pcca (no. 54), a = 13.9484(12) Å, b = 18.5280(16) Å, c = 7.0215(8) Å, V = 1814.6(3) Å^3^, Z = 8, μ(MoKα) = 0.562 mm^−1^, Dcalc = 1.591 g/cm^3^, 12,574 reflections measured (6.8° ≤ 2Θ ≤ 52.6°), 1842 unique (Rint = 0.0496, Rsigma = 0.0281) which were used in all calculations. The final R1 was 0.0387 (I > 2σ(I)), and wR2 was 0.0900 (all data).

Crystal Data for **1b** (C_9_H_9_O_4_NK; M = 234.27 g/mol): monoclinic, space group P2_1_/c (no. 14), a = 18.879(3) Å, b = 6.2395(6) Å, c = 8.7555(9) Å, β = 99.765(12)°, V = 1016.4(2) Å^3^, Z = 4, μ(CuKα) = 4.567 mm^−1^, Dcalc = 1.531 g/cm^3^, 3619 reflections measured (9.5° ≤ 2Θ ≤ 117.6°), 1350 unique (Rint = 0.0320, Rsigma = 0.0421 which were used in all calculations. The final R1 was 0.0615 (I > 2σ(I)) and wR2 was 0.1854 (all data).

### 4.5. Moisture and Thermal Analysis

TGA curves were recorded using a LINSEIS STA PT1600 at a heating rate of 10 °C/min under an argon atmosphere over a 25 °C to 800 °C temperature range. Moisture analysis was performed on a Radwag MA 50/1.R.WH.

### 4.6. Mass Spectrometry

Mass spectrometry employed an API 4000 QTRAP tandem mass spectrometer detector equipped with an electrospray ionization (ESI) source (Applied Biosystems/MDS SCIEX, Foster City, CA, USA).

## Figures and Tables

**Table 1:** Survey of synthesis methods of 1 *N*-[(2-hydroxyphenyl)methylene]glycine found in the literature.

Reference	Solvent	Reaction Time	Temperature	Product Form (Isolation Method)	Yield
[15]	Water/EtOH 2:1	3 h	reflux	Solid (filtered, washed ethanol and ether, recrystallization ethanol:propanol mixture)	66%
[16]	Water + KOH/MeOH 1:1.5	1 h	r.t.	Not Separated (used as a solution)	not specified
[17]	Water + NaOH/EtOH 1:3	2 h	10 °C	Solid (filtered, recrystallization methanol:ether mixture)	58%
[18]	EtOH + KOH	1 h	50 °C	Solid (extracted with diethyl ether, and filtered off)	63%
[19]	EtOH + KOH	not specified	RT	Solid (extracted with diethyl ether, and filtered off)	63%

**Table 2:** Hydrogen bonding geometry (Å, °) for structures **1a** and **1b**.

Compound	D–H···A	d(D–H)	d(H···A)	d(D···A)	< (D–H···A)
1a	N1-H1···O1	0.86	1.94	2.621(2)	135.3
	N1-H1···O3	0.86	2.29	2.668(2)	107.1
1b	N1-H1···O1	0.88	1.92	2.606(5)	133.8
	N1-H1···O2W	0.88	2.44	3.017(8)	124
	C8-H8A···O3A ^i^	0.99	2.47	3.318(6)	143
	O2W-H2WA···O1 ^ii^	0.87	1.98	2.670(7)	135.4
	O2W-H2WB···O1	0.87	2.59	3.337(7)	143.4
	O2W-H2WB···O2 ^iii^	0.87	2.47	2.892(7)	110.4

i: x, −y + 3/2, z − 1/2; ii: x, −y + 5/2, z − 1/2; iii: x, y + 1, z.

## Supplementary Materials

The following are available online, ^1^H-NMR, ^13^C-NMR, MS, UV-Vis spectra and TGA analysis.
