# Supplementary material for: Unprecedented Water Effect as a Key Element in Salicyl-Glycine Schiff Base Synthesis
Source: Molecules. 2020 Mar 10;25(5):1257. doi: 10.3390/molecules25051257 (PMC7179422; doi:10.3390/molecules25051257)
Supplement: Supplementary file 1 [file molecules-25-01257-s001.zip › Kuznik-SupplMat-2020304.docx]

**Unprecedented water effect as a key element in salicyl-glycine Schiff base synthesis**

Karolina Bakalorz^a^, Łukasz Przypis^a^, Mateusz M. Tomczyk^a^, Maria Książek^b^, Ryszard Grzesik^c^, Nikodem Kuźnik^a^*

^a^ Faculty of Chemistry, Department of Organic Chemistry Bioorganic Chemistry and Biotechnology, Silesian University of Technology, B. Krzywoustego 4, 44-100 Gliwice, Poland

^b^ Department of Physics of Crystals, Institute of Physics, University of Silesia, 75 Pułku Piechoty 1, 41-500 Chorzów, Poland

^c^ Department of Research and Innovations, Grupa Azoty ZAK S.A. Mostowa 30 A, 47-220 Kędzierzyn-Koźle, Poland

* nikodem.kuznik@polsl.pl, +48 694220077

Figure S1: ^1^H NMR spectrum of 1.

Figure S2: ^1^H NMR spectrum of 1, zoomed.

Figure S3: ^13^C NMR spectrum of 1.

Figure S4: MS spectrum of 1.

**Synthesis of potassium *N*-[(2-hydroxyphenyl)methylene]glycinate “without water"**

Glycine (5.00 g, 66.67 mmol) and potassium hydroxide (3,70 g; 66,67 mmol) was added to a Schlenk tube and was then evacuated and backfilled with nitrogen. Then, it was mixed and the temperature was raised to 70 ^o^C, leading to melted potassium glycinate. Next, freshly distilled salicylaldehyde (6,9 mL; 66,67 mmol) was added slowly with a syringe, under a N_2_ atmosphere (Schlenk line) (Fig. S5. A). The resulting mixture was stirred for:

1. 30 min and cooled to room temperature. In addition, TLC analysis showed the presence of large amounts of salicylaldehyde. Then, it was evaporated under reduced pressure to give semi-solid residue (8,54 g). (Fig S5.B).


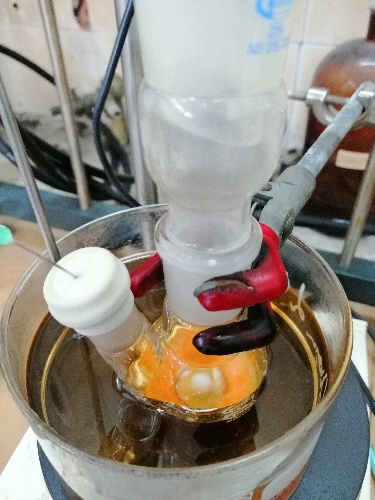

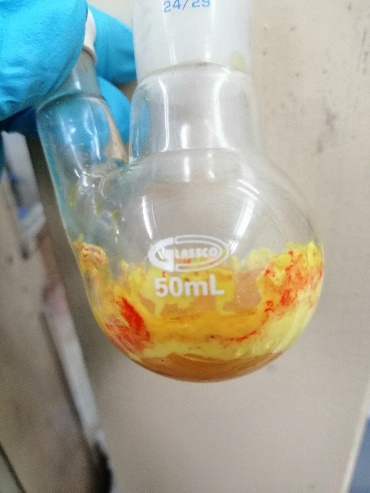

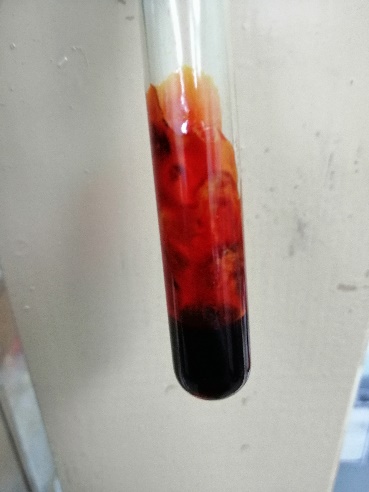


**C**

**B**

**A**

Figure. S5. Progress of the reaction, experiment “without water”: A – After added salicylaldehyde; B – After 30 minutes; C – After 1 hour

**B**

Figure. S6. ^1^H NMR spectrum of from experiment “without water”, after 30 minutes

1. A 1 hour and cooled to room temperature. The reaction mixture was worked up to give a brown oil-solid system (8,11 g). (Fig S5.C). In addition, TLC analysis showed the presence of many inseparable products.

**C**

Figure. S7. ^1^H NMR spectrum of from experiment “without water”, after 1 hour.

**Synthesis of potassium *N*-[(2-hydroxyphenyl)methylene]glycinate in anhydrous conditions**

Glycine (1.00 g, 13,33 mmol) and potassium hydroxide (740 mg; 13,33 mmol) was added to a Schlenk tube and was then evacuated and backfilled with nitrogen and anhydrous methanol was added (10 mL). Then, it was mixed and raised temperature to 70 ^o^C. Next, freshly distilled salicylaldehyde (1,38 mL; 13,33 mmol) under a N_2_ atmosphere (Schlenk line) (Fig. A). Then yellow solid was precipitated immediately (Fig. S8 D). The TLC analysis showed the presence still large amounts of salicylaldehyde. After 6 hours, it was observed full conversion of aldehyde. Then resulting mixture was cooled to room temperature (Fig.S8 E) and it was evaporated under reduced pressure to give orange oil (1,79 g).


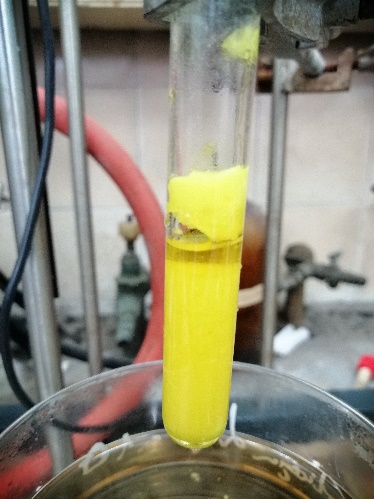

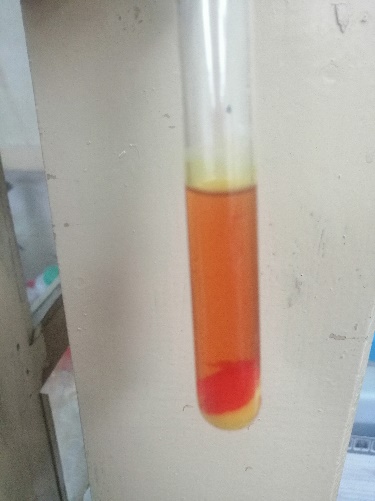


**E**

**D**

Figure. S8. Progress of the reaction, experiment “anhydrous conditions”: D – After added salicylaldehyde; E – After 6 hours

**E**

Figure. S9. ^1^H NMR spectrum of from experiment “anhydrous conditions”, after 6 hours.

UV-VIS spectra (all measurements were made in MeOH)

Figure S10: Compound **1a** + 10% (molar mass) FeCl_3_

Figure S11: Compound **1a** + 10% (molar mass) CuCl_2_

Figure S12: Salicylaldehyde + 1 equiv KOH

Figure S13: Compound **1a** + 1 equiv FeCl

Figure S14: Compound **1a** + 1 equiv CuCl_2_

Figure S15: Comparison of compound **1a** and substrates with FeCl_3_

Figure S16: Compound **1a**

Figure S17: Compound **1a** + 1 equiv of water

Figure S18: Compound **1a** + 10 equiv of water

**Synthesis of 1a-Cu complex in DMF**

1 mmol (1 equiv, 217 mg) of anhydrous ligand 1a, 0.5 mmol (0.5 equiv, 125 mg) of CuSO_4_·5H_2_O were suspended in 2.5 ml of anhydrous DMF, heated to 80 ^o^C and stirred under that temperature for 1 h. During the stirring reaction mixture turned from yellow to green and deep blue. After 30 minutes of stirring blue precipitate was formed. Upon completion of the reaction, it was cooled to room temperature and 10 ml of diethyl ether were added which created even more precipitate. The suspension was filtered, the precipitate was washed with small portions of toluene and diethyl ether and dried on a rotary evaporator under reduced pressure to give 1a-Cu complex as a light blue powder with 81% yield (240 mg, 0.48 mmol). MS for 1a-Cu complex [C_9_H_7_CuNKO_3_]^+^ calculated: 278,9 (100,0%), 280,9 (44,6%), 280,9 (7,2%); found: 278,9 (100,0%); 280,9 (54,0%)

Figure S19: MS spectrum of a 1a-Cu metal complex obtained from DMF

Figure S20 TGA curves of compounds: 1a – dried after the reaction, 1b – hydrated form obtained from 1a, while 1a obtained by drying hydrated form 1b.


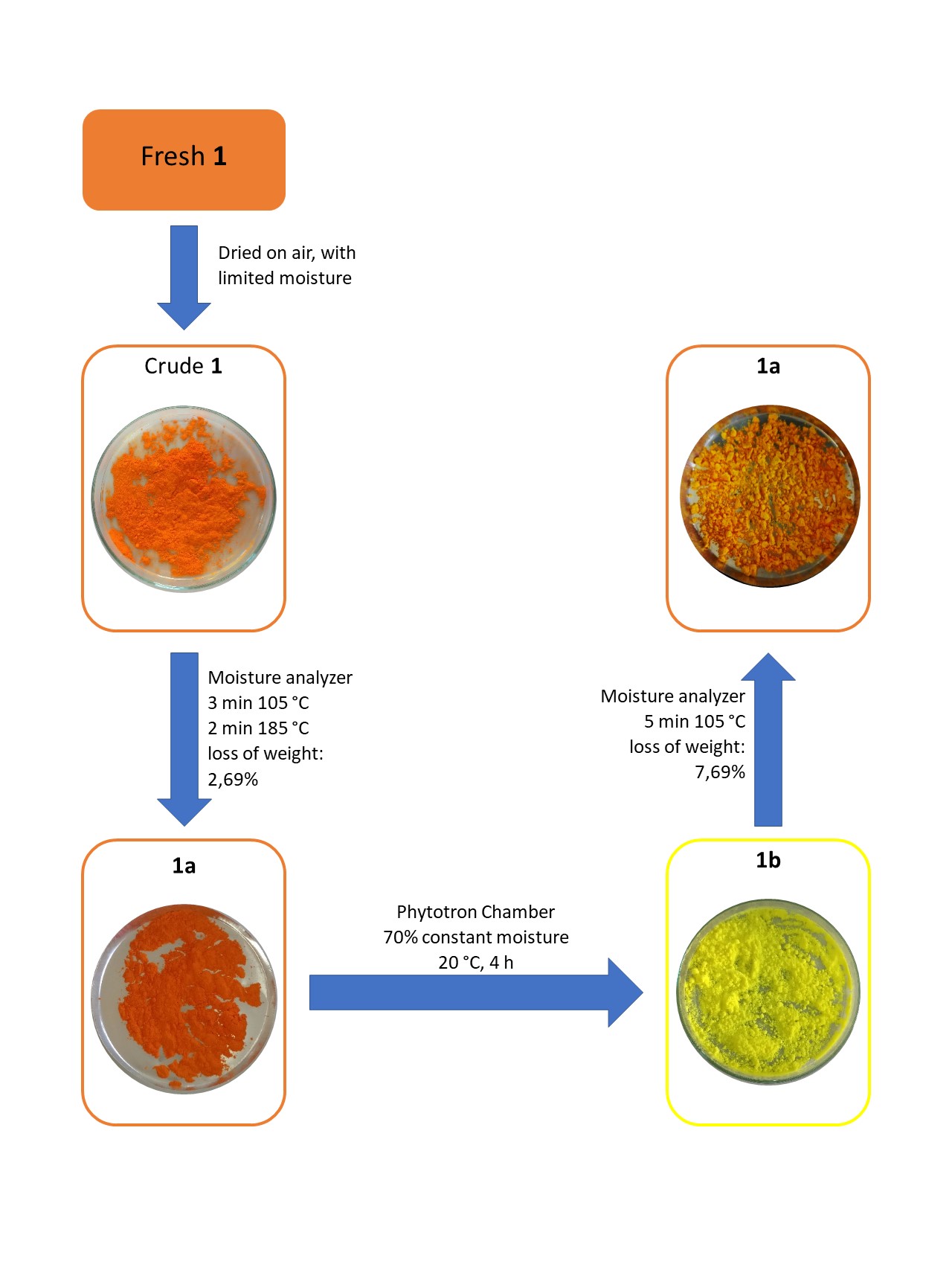


Figure S21: Moisture analyzer results

The characteristics of the fingerprint plots and Hirshfeld surface

The intermolecular interactions in compounds **1a** and **1b** are shown on the two-dimensional (2D) fingerprint plots in Figure S22 obtained using Crystal Explorer program. For the compound **1a**, the bottom part of the plot and very short bottom spike correspond the O···H/H···O interactions, while the central part of the plot with light colors corresponds to the O···K/K···O interactions. The H···H interactions are also reflected in the central part. The area corresponding to the H···C/C···H interactions is placed in the upper left part of the plot. For the compound **1b**, the upper and bottom part of the plot corresponds to the O···H/H···O interactions. They are also reflected by the part of the upper spike and the bottom spike. Similarly to **1a**, the central part of the plot with light colors also corresponds to the O···K/K···O interactions. The central part of the plot and the middle spike corresponds to the H···H interactions. The area corresponding to the H···C/C···H interactions is placed in the upper left and upper right parts of the plot.

| a) | 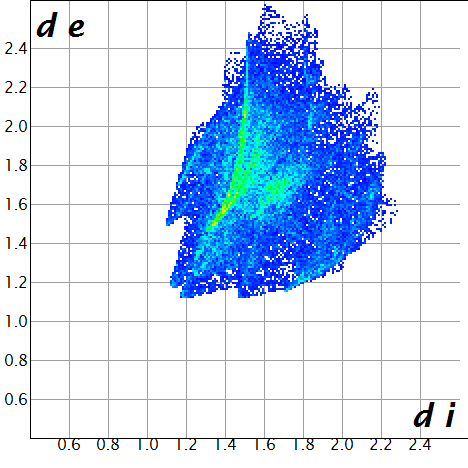 | b) | 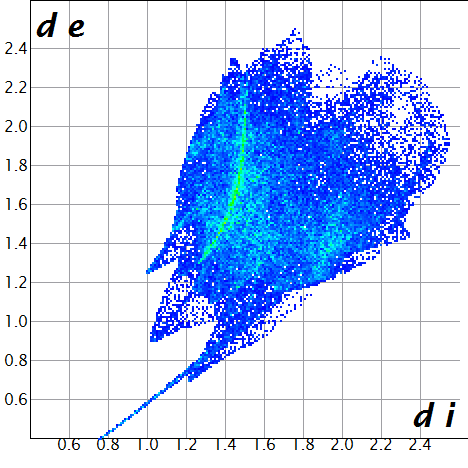 |
| --- | --- | --- | --- |

**Figure S22.** Two – dimensional fingerprint plots for compounds 1a (a) and 1b (b).

The most important types of interactions (O···H/H···O, O···K/K···O, H···H, H···C/C···H) are summarized in Table S1 along with their percentage contribution to the Hirshfeld surface.

**Table S1.** Selected atom-atom intermolecular interactions and their contributions to the Hirshfeld surface in 1a and 1b.

| Type of interactions | Contribution to the surface [%] | |
| --- | --- | --- |
|  | 1a | 1b |
| O···H/H···O | 10.7 | 14.4 |
| O···K/K···O | 16.7 | 10.6 |
| H···H | 41.8 | 37.9 |
| H···C/C···H | 18.1 | 29.0 |
